# Implications of ZnO Nanoparticles and S-Nitrosoglutathione on Nitric Oxide, Reactive Oxidative Species, Photosynthetic Pigments, and Ionomic Profile in Rice

**DOI:** 10.3390/antiox12101871

**Published:** 2023-10-17

**Authors:** Isabella Martins Lourenço, Bruna Moreira Freire, Joana Claudio Pieretti, Roberta Albino dos Reis, Nicolas Martins Soares, Marcelo da Luz Santos, Bruno Lemos Batista, Amedea Barozzi Seabra, Camila Neves Lange

**Affiliations:** Center for Natural and Human Sciences (CCNH), Federal University of ABC (UFABC), Santo André 09210-580, SP, Brazil; isabella.lourenco@ufabc.edu.br (I.M.L.); bruna.freire@ufabc.edu.br (B.M.F.); joana.pieretti@ufabc.edu.br (J.C.P.); roberta.reis@ufabc.edu.br (R.A.d.R.); n.martins@ufabc.edu.br (N.M.S.); marcelo.luz@ufabc.edu.br (M.d.L.S.); bruno.lemos@ufabc.edu.br (B.L.B.); amedea.seabra@ufabc.edu.br (A.B.S.)

**Keywords:** zinc oxide nanoparticles, nitric oxide donor, rice, mineral profile, photosynthetic pigments

## Abstract

Zinc is an important nutrient for several plants and humans. Nitric oxide (NO) is a free radical that is important to biological processes that mediate the growth and mitigation of biotic and abiotic stresses in plants. The present study investigated the enzymatic and photosynthetic profile and the accumulation of macro- and microelements in rice plants (*Oryza sativa* L.) that received foliar treatments of zinc oxide nanoparticles (ZnO NPs), nitric oxide donor (GSNO), and the association of both (GSNO–ZnO NPs). Zinc concentration in rice husks increased by 66% and 68% in plants treated with ZnO NPs and GSNO–ZnO NPs, respectively. The GSNO treatment caused an increase of 25% in the Fe concentration in the rice grains. Only a small disturbance of the antioxidant system was observed, with increases in H_2_O_2_, S-NO, and NO_2_^−^, mainly in the group treated with GSNO–ZnO NPs; however, the disturbance did not affect the yield, the growth, or vital processes, such as as photosynthetic pigments production. There was an increase in chlorophyll B of 290% and an increase in chlorophyll A of 187% when ZnO NPs was applied. GSNO–ZnO NPs increased chlorophyll B by 345% and chlorophyll A by 345%, indicating that the treatments GSNO, ZnO NPs, and GSNO–ZnO NPs reduced possible oxidative stress and helped as protective treatments.

## 1. Introduction

Food security is an issue that is identified as one of the main challenges in maintaining the sustainability of human beings on the planet. More than two billion people in the world have micronutrient deficiencies [1]. Rice is the staple food for half of the world’s population, and it is considered an important food source in several countries of the world [2]. In rice grains, essential elements such as copper (Cu), zinc (Zn), and magnesium (Mg) are present and are essential for humans and other living beings, as they are related to homeostasis. These elements are important components of molecules and enzymes involved in oxygen transport, hormonal activity, metabolism control, signaling pathways, and free radical scavenging.

Dietary zinc deficiency is a well-studied fact, particularly in developing countries [3,4], and it affects approximately one-third of the world’s population—primarily children and pregnant women [5]. Zinc is an essential mineral that is widely involved in bodily functions, including cell metabolism, and it is necessary for the proper functioning of dozens of bodily enzymes. It is also vital for the immune system, protein synthesis, DNA synthesis, and cell division [4,6]. Zinc deficiency affects 50% of arable soils and is recognized as the most critical micronutrient deficiency in crops [7]. Tropical soils are largely acid soils with high base saturations and high phosphate contents; when they are subjected to prolonged flooding or inundation, zinc deficiency in crops can result [3]. This deficiency not only limits crop productivity, but also reduces grain yield and quality. Therefore, declining overall soil quality poses a major challenge in improving zinc content in grains and soil quality is a high priority area of research [8,9].

The biofortification of crops is being introduced in several countries as a strategy to reduce micronutrient deficiencies [10].

Nanoparticles (NPs) are materials that have unique physical–chemical properties, due to their reduced size [11]. In the nanometer scale, the electronic, magnetic, optic, catalytic, biomedical, and thermodynamic properties differ drastically from those of bulk materials. These physicochemical properties are dominated by the materials’ sizes, shapes, dimensionality, crystallinity, structures, compositions, etc. [12].

The aim of using nanomaterials in agriculture is to decrease the amount of hazardous chemicals, minimize nutrient losses in fertilization, and increase yield through pest and nutrient management [13]. Thus, zinc nanoparticles can be a suitable alternative to increase the concentration of zinc in rice grains. The lack of this microelement leads to dysfunctions in the enzymatic and catalytic systems and in the factors that regulate plant growth, consequently reducing the yield of grains and the nutritional value of plants [14,15]. Furthermore, several studies have demonstrated that the use of ZnO NPs has positive effects on plants of different species, in terms of improving nutritional content, the photosynthetic pigment rate, fruit quality, and activation of the defense system [16]. However, both positive and negative effects of NPs on plants have been reported, and some reports have confirmed that NPs can induce phytotoxicity and have a negative effect on plant development and growth [13,17]. 

The free radical nitric oxide (NO) may act as an agent to mitigate the drawbacks of nanoparticles’ application. This molecule plays important roles in biological systems, due to its diffusive nature and its ability to participate in intercellular communication, and it plays an important role in plant physiology [18,19,20]. Recent studies have shown that the application of exogenous NO donors in plants is responsible for their development and growth, as NO breaks seed dormancy and promotes seed germination, root growth, flowering, and growth regulation of the pollen tube; NO is also involved in photosynthesis, in plant metabolism and cell death, in controlling the opening and closing of stomata, and in responses to abiotic stresses (such as water deficits, salinity, and contamination by metals) and biotic stresses (such as infections caused by insect pests, fungi, bacteria, viruses, and nematodes) [18,19,21,22,23]. A previous study showed that a combined administration of metallic NPs and S-Nitrosoglutathione (GSNO), an NO donor, enhanced important physiological, biochemical, and nutritional parameters in plants [24].

Despite considerable progress in recent years in the application of nanoparticles in agriculture, there are gaps in knowledge, especially when considering information regarding the use of these promising materials in the biofortification of cereals and in the mitigation of their potential toxic effects by an NO donor. This study aims to evaluate the foliar application of an NO donor (GSNO) associated with zinc nanoparticles (ZnO NPs) for grain biofortification, as well as to observe the alteration of the interrelationship of macro- and microelements and biochemical parameters under the influence of the imposed variables. To the best of our knowledge, this is the first report that demonstrates the effects of ZnO NPs combined with GSNO in rice.

## 2. Materials and Methods

### 2.1. Chemicals 

High-purity deionized water (MS2000, Gehaka, São Paulo, Brazil, resistivity at 18.2 MΩ cm) was used to prepare samples, solutions, and standards. All samples and prepared solutions were stored in 15 mL or 50 mL Falcon-type polypropylene conical flasks. Zinc acetate dihydrate (Zn(CH_3_COO)_2_·2H_2_O), glutathione (GSH), sodium nitrite (NaNO_2_), sulfuric acid (H_2_SO_4_), Triton X-100, ETDA (0.1 mmol L^−1^), ascorbic acid (0.5 mmol L^−1^), nitrotetrazolium chloride blue (NBT), methionine (13 mmol L^−1^), and riboflavin were obtained from Sigma–Aldrich (St. Louis, MO, USA). Potassium phosphate (KH_2_PO_4_), hydrogen peroxide (H_2_O_2_), methionine (13 mmol L^−1^), riboflavin, and nitric acid (HNO_3_) were obtained from Labsynth (Diadema, São Paulo, Brazil).

### 2.2. Synthesis and Characterization of ZnO NPs 

Zinc oxide nanoparticles (ZnO NPs) samples were prepared using the hydrothermal method with oven heating [11]. The hydrothermal synthesis carried out in this study was used in a homogeneous aqueous medium and with temperature elevation greater than 25 °C, applied to facilitate the process of crystallization of ZnO NPs. Conditions such as high temperature and pressure can lead to recrystallization processes, allowing a certain grain size control over the stages of nucleation, growth, and the increase in crystallinity, generating a solid and moldable material [25,26]. The recrystallization process of ZnO NPs within a hydrothermal reactor initially originates at the critical point threshold of water [27]. Thus, the objective of this mechanism is to control the uniformity of nucleation and the aging of nanocrystallites. Consequently, the high temperature and pressure help to reduce particle aggregation, a phenomenon that may not be possible in other synthetic methods [26,28,29].

**Figure 1:**
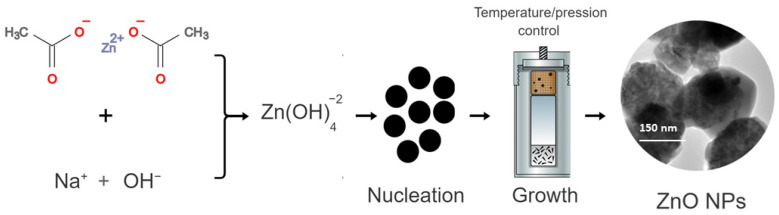
Schematic representation of the synthesis of ZnO by the hydrothermal method.

ZnO NPs synthesis began with the formation of zinc hydroxide species, in a condition of a basic environment—herein, in the presence of NaOH. Thereafter, the zinc hydroxide species led to the formation of crystalline nuclei, starting the crystal growth process [30]. The disturbance of the system, with increased pressure and temperature added to the hydrothermal reaction resulted in the recrystallization of ZnO, directly influencing the growth of anisotropic crystals and crystallization in hexagonal ZnO NPs [31]. Figure 1 shows a schematic representation of this synthesis.

Initially, an aqueous solution of zinc acetate dihydrate (Zn(CH_3_COO)_2_·2H_2_O) was prepared by weighing a mass of 2.195 g of zinc acetate in 30 mL of deionized water. Then, 30 mL of NaOH (1 mol L^−1^) was slowly added to the zinc acetate solution under constant stirring. After the end of the dripping, the final suspension with a milky white color was transferred to the hydrothermal apparatus at 170 °C for a period of 10 h. Then, the obtained material was washed with absolute ethyl alcohol and deionized water three times, and subsequently dried in an oven for 2 h at 60 °C.

The synthesized ZnO NPs were characterized by different techniques. Their hydrodynamic size, polydispersity index, and surface zeta potential were analyzed by dynamic light scattering (DLS) and electrophoretic mobility (Nano ZS Zetasizer, Mavern Instruments Co., Worcestershire, UK). The surface morphology and size distribution of solids were investigated by transmission electron microscopy (TEM, JEM-2100 Plus, 200 kV, JEOL, Peabody, MA, USA). For TEM analysis, diluted suspensions of the materials were dropcast on carbon-coated grids and air-dried at room temperature (25 °C). The crystalline structure was characterized by X-ray diffraction (XRD) (PANalytical, model: Xpert Pro, Almelo, The Netherlands).

### 2.3. Synthesis of GSNO 

GSNO was synthesized, according to the method of Pellegrino et al. [32]. Briefly, 13 g of reduced L-GSH were weighed and solubilized in 34 mL of HCl (1 mol L^−1^), followed by the addition of 3.06 g of NaNO_2_. The mixture was kept in an ice bath, under constant magnetic stirring, and protected from light for 40 min. Then, acetone was added to aid in the precipitation of GSNO. The precipitate obtained with the consistency of a pinkish cream was washed and filtered with the aid of a vacuum filter with a 0.45 μm cellulose membrane (Millipore). After filtration, the GSNO obtained was freeze-dried for 24 h and stored at −20 °C, protected from light. The material obtained was lyophilized for 24 h and stored at −20 °C, protected from light. UV-visible spectrometry (model 8454, Agilent, Palo Alto, Santa Clara, CA, USA) was used to confirm the formation of the GSNO by the detection of S-NO bonds at 336 nm and 545 nm. 

### 2.4. Pot Experiments 

Rice plants (*Oryza sativa* L.) were cultivated between November 2021 and April 2022 in a greenhouse with controlled humidity and temperature (respectively, 50% to60% humidity and 25 °C). Rice seeds (BRS PAMPA variety) provided by Embrapa were planted in individual 8 L plastic pots with 70% organic soil and 30% topsoil. Then, the plants were cultivated in flooded conditions. The treatments were applied by foliar spraying and were divided as follows: (i) surfactant Triton X-100 0.1% (control group); (ii) ZnO NPs + Triton X-100 0.1% (group Z); (iii) nitric oxide donor GSNO (group G) + Triton X-100 0.1%; and (iv) ZnO NPs + GSNO (GZ group) + Triton X-100 0.1%. The 4 groups were divided to contain 4 replicates that were randomly distributed in blocks composed of 2 rows of 17 m spaced 0.30 m apart.

A suspension containing only ZnO NPs was sonicated for 30 min before application. The treatments were allocated in manual sprayers and a volume of 70 mL per day was applied in each pot. The frequency of exposure per group was twice a week for four weeks. The final added concentration of ZnO NPs was 100 µg mL^−1^ and the amount of GSNO was 100 mM, which was defined based on other studies carried out by our research group [33]. The pots of each group remained in the greenhouse until panicle formation and grain maturation.

The grains were collected after complete maturity (after approximately 6 months of cultivation). The shoots were cut 2 cm above the soil surface and the plant parts (roots, shoots, and leaves) were washed. A portion of the fresh part of each tissue was separated and kept for conservation and later analysis—either dried in an oven at a temperature of 40 °C for 72 h or stored in a freezer at –80 °C. After complete drying, the shoots, roots, and grains were weighed to determine the dry weight.

### 2.5. Biochemical Indicators

The following subsections describe the biochemical parameters measured. A UV–Vis spectrophotometer (8454, Agilent, Palo Alto, CA, USA) was used to determine all the absorbances.

#### 2.5.1. Enzymatic Activity

For the determination of the enzymatic activity of ascorbate peroxidase (APX), superoxide dismutase (SOD), and peroxidases (POD), 100 mg of fresh plant samples previously stored in a −80 °C was used. Leaf and root samples were macerated in liquid nitrogen and 1 mL of extraction buffer was added, consisting of EDTA (1 mmol L^−1^) and PVPP (2% *m*/*v*) in potassium phosphate buffer (0.1 M pH 6.8). The macerated extract was sonicated (60 kHz) for 10 s and centrifuged for 10 min (15.644× *g*). The supernatant was used to designate the enzymatic activity [33]. 

For APX, an aliquot of the prepared extract (25 µL) was mixed in 1.45 mL of a solution containing EDTA (0.1 mmol L^−1^), potassium phosphate buffer (50 mmol L^−1^, pH 7.0), and ascorbic acid (0.5 mmol L^−1^). Then, 25 µL of H_2_O_2_ (30 mmol L^−1^) was added and, after 2 min, the absorbance was measured at 290 nm [33]. Lambert Beer’s law (ɛ = 2.8 L mmol^−1^ cm^−1^) was used to calculate enzyme activity [34].

The SOD was determined by adding an aliquot of 40 µL of the extract in 1.96 mL of potassium phosphate buffer (50 mmol L^−1^, pH 7.8) containing riboflavin (2 µmol L^−1^), EDTA (0.1 mmol L^−1^), NBT (75 µmol L^−1^), and methionine (13 mmol L^−1^). This reaction mixture was exposed to photosynthetic active radiation equivalent to 300 μmol m^2^ s^−1^ using a 60 W fluorescent lamp. After 10 min, the absorbance of the mixture was measured at 560 nm. A mixture identical to the first was kept in the dark and then, used as a blank standard for each sample. Another mixture containing only distilled water in place of the extract was irradiated and used as a control sample. For the calculations, one SOD unit was considered as the amount of enzyme that was required to inhibit NBT reduction by 50% [35].

To quantify POD activity, a 10 µL aliquot of the extract was mixed in 1.79 mL of phosphate buffer (20 mmol L^−1^, pH 6.8) containing H_2_O_2_ (20 mmol L^−1^) and pyrogallol (20 mmol L^−1^). After 1 min of reaction, the absorbance was measured at 420 nm [33]. Enzyme activity was calculated using Lambert Beer’s law (ɛ = 2.47 L mmol^−1^ cm^−1^) [36].

#### 2.5.2. S-Nitrosothiols, NO_2_^−^, and H_2_O_2_ Determinations

For the quantification of nitrite (NO_2_^−^), S-nitrosothiols (S-NO), and hydrogen peroxide (H_2_O_2_), 100 mg of the fresh leaf and root samples were weighed and frozen at −80 °C. Samples were macerated by adding 1 mL of N-ethylmaleimide solution (NEM, 5 mmol L^−1^), followed by sonication (60 kHz) for 10 s and centrifugation (15.644× *g*) for 10 min. Aliquots of 20 µL of the supernatant were read in duplicate using a WPI TBR4100/1025 ammeter (World Precision Instruments Inc., Sarasota, FL, USA) and an H_2_O_2_ (ISO-HPO^−2^) or NO (ISO-HPO^−2^) sensor for analysis. KI/H_2_SO_4_ (100 mmol L^−1^, 10 mL), CuCl_2_ (100 mmol L^−1^, 10 mL) and PBS (100 mmol L^−1^, 10 mL) solutions were used for the quantification of NO_2_^−^, S-NO, and H_2_O_2_. The results obtained were compared with calibration curves previously performed with NaNO_2_ (to quantify NO_2_^−^), GSNO (to quantify S-NO), and H_2_O_2_ (to quantify hydrogen peroxide) [32,37].

#### 2.5.3. Pigment Contents

The determination of photosynthetic pigments, such as chlorophyll and carotenoids, was analyzed only in samples of rice leaves. The control group and the ZnO NPs, GSNO, and GSNO–ZnO NPs groups contained 3 replicates, each distributed randomly. Subsequently, a mass of approximately 50 mg was macerated and swollen in 10 mL of ethanol (99% *v*/*v*). The samples were wrapped in aluminum foil and stored at 4 °C for 4 days. On the fifth day, the pigments were determined at lengths of 470, 649, and 665 nm. The concentrations were calculated according to the methodology of Lichtenthaler & Wellburn [38].

### 2.6. Elemental Determination

Of the collected rice grains, those of about 5–6 g (dry mass) were manually husked; then, the husks and grains were stored separately. Subsequently, the grains and husks were ground and sieved in a pore sieve (<250 μm). Around 150 mg of samples of the husks and grains were pre-digested with 2 mL of subdistilled HNO_3_ for 48 h. The samples were heated in a digester block at 95 °C for 4 h, as described by Paniz et al. [39]. After cooling, the digested samples were diluted with 30 mL of ultrapure water. 

Shoot samples were predigested for 48 h with 1.5 mL of subdistilled HNO_3_, while roots were predigested with 1 mL of subdistilled HNO_3_. Then, they were taken to the digester block for 4 h at 95 °C. When cooling the samples, 20 mL of ultrapure water was added to the shoot samples and 10 mL of ultrapure water was added to the root samples.

The elements present in dry grains, husks, shoots, and roots were determined by inductively coupled plasma mass spectrometry (Agilent 7900 ICP-MS, Hachioji, Japan). A stock solution containing all elements (10 mg L^−1^) (Perkin Elmer, Waltham, MA, USA) was used to prepare the calibration curve, according to the methodology of Paniz et al. [39]. To ensure the accuracy and reliability of the results obtained, certified reference materials—NIST 1568b (rice flour), NIST 1640a (natural water), CRM agro AR_01/2015 USP/Embrapa, CRM agro C1001a (brown rice), and NIST1573a (tomato leaves)—were analyzed. The results of the analysis of the CRMs were statistically consistent with the certified values.

### 2.7. Translocation Factor

The translocation factor (TF) of each element was defined as the ratio of the element concentration in the shoots to that in the roots. 

### 2.8. Statistical Analysis 

Statistical analyses were performed by analysis of variance (ANOVA), at *p* ≤ 0.05 level of significance.

## 3. Results and Discussion

### 3.1. Synthesis and Characterization of ZnO NPs

**Figure 2:**
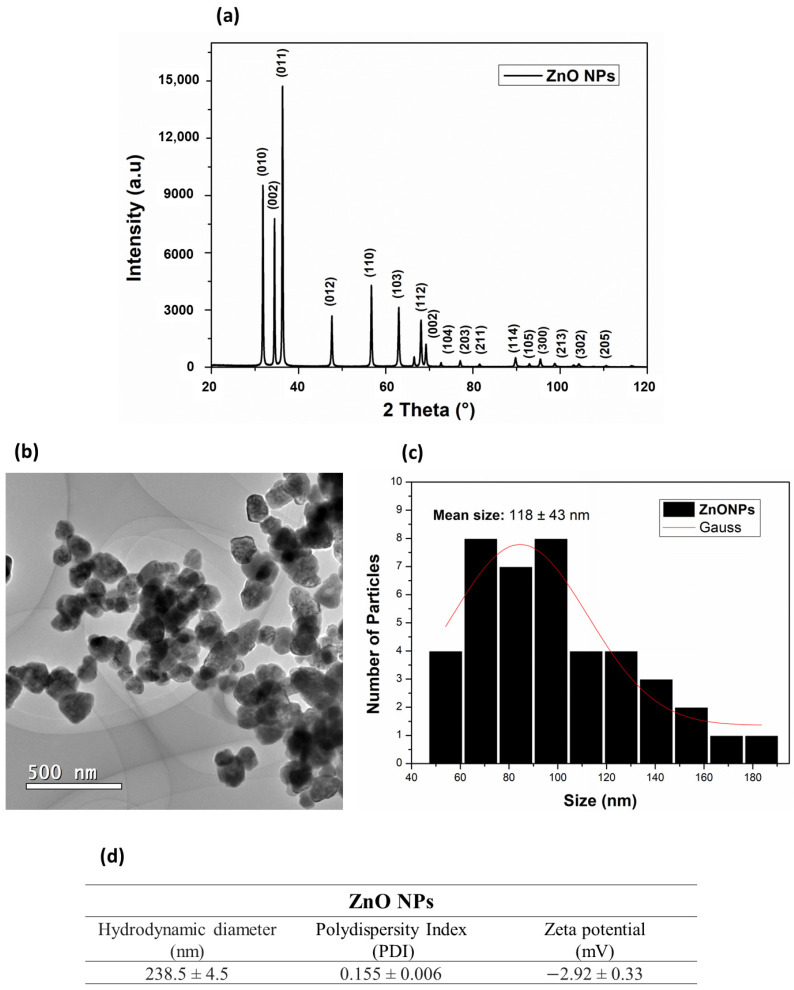
(**a**) Diffractogram of ZnO NPs; (**b**) representative TEM images of ZnO NPs; (**c**) histogram for particle-size distribution; (**d**) hidrodynamic diameter, polydispersity index, and zeta potential of ZnO NPs.

An X-ray diffraction (XRD) technique was used to verify the crystalline structure of the ZnO NPs. The results (Figure 2a) showed phase reflections for the crystalline structure in the deflection planes (010), (002), (011), (012), (110), (103), (112), (002), (104), (203), (114), (105), (300), (213), (302), and (205). The 2θ diffraction peaks of 31.07°, 34.52°, 36.45°, 47.73°, 56.73°, 63.06°, 68.01°, 69.35°, 72.59°, and 76.98° corresponded to the cubic structure of ZnO NPs (JSPDS card No. 36-1451) (Figure 2a). The reflections presented in the XRD phases for the ZnO NPs were fitted with the Gaussian distribution, and the ZnO NPs grain-size value was estimated, using the Debye–Scherrer equation for the largest prominent peak, at 2θ (36.32°), resulting in grain sizes of 39 nm. These results were in common agreement with previously XRD-reported data for the ZnO NPs average grain sizes, ranging from 7–16 nm [40] to 15.8 nm [41] to sizes below 60 nm [42] and even above 50 nm [43].

The morphology and sizes of the ZnO NPs in the solid state were observed by transmission electron microscopy (TEM) (Figure 2b). A hexagonal shape and an average size distribution of 118 ± 43 nm (Figure 2c) were observed. As in the present study, Rai et al. [44] synthesized ZnO NPs by the hydrothermal method, ranging from 100 nm to 150 nm in the solid state. Rai et al. also reported that the size and morphology of these nanoparticles were closely related to the zinc precursor salt and the temperature used to obtain them. Vlazan et al. [45] and Aneesh et al. [40] reported that ZnO NPs synthesized by the hydrothermal method can present morphologies ranging from spherical to hexagonal to irregular in the presence of agglomerated particles. Although different zinc salts (Zn(NO_3_)_2_·6H_2_O, Zn(CH_3_COO)_2_·2H_2_O, and ZnCl_2_) produce hexagonal nanoparticles in hydrothermal synthesis, some of them, such as zinc chloride, may contain impurities that produce a stable by-product (simonkolleite), which is considered an impurity during the synthesis of ZnO [46,47,48]. The morphology and the sizes of NPs are also influenced by the type of precursor salt, because different salts present distinct kinetics decomposition [49]. 

X-ray diffraction (XRD) and transmission electron microscopy (TEM) are complementary techniques for nanoparticles characterization. TEM provides information on the size and the shape of subnanoclusters and, in this analytical technique, the size distribution was calculated considering the Gaussian standard deviation. However, Figure 2c shows a nonsymmetrical size-distribution profile, indicating particles agglomeration. XRD can only detect the crystalline portion of a particle. These differences may arise due to different sensitivities, measurement conditions, or the limitations of each technique [50]. 

DLS information is provided in Figure 2d. The observed hydrodynamic diameter of ZnO NPs was 238.5 ± 4.5 nm and their PDI was 0.155 ± 0.006. The zeta potential obtained by DLS was −2.92 ± 0.33 mV. It is known that PDI is associated with particle dispersion, and the lower this value, the more homogeneous the NPs [51]. 

Previous studies reported a size-dependent mechanism of nanoparticles uptake into the body of a plant [52,53]. Compared to bulk materials, the smaller size of NPs provides higher reactivity, solubility, and bioavailability [54]. Small size NPs are easily transferred within the plant tissue and may have a higher level of accumulation, but they may also cause more toxicity and they can induce DNA damage; therefore, the right dosage of appropriately sized NPs promotes absorption by plants [55]. It is difficult to determine the optimal design of NPs for rice crops, since many of the previous studies were performed under hydroponic conditions at seedling levels and during short-term periods [56].

### 3.2. Growth Attributing Characters

The effects of foliar application of ZnO NPs, GSNO, and GSNO–ZnO NPs on the growth and productivity of rice are shown in Table 1. The plants treated with GSNO alone or GSNO combined with ZnO NPs presented a higher number of panicles and a lower shoot dry mass than the control plants. Root dry mass was not affected by any treatment. Interestingly, the exposure of rice plants to ZnO NPs or GSNO alone significantly increased grain productivity, by 45 and 35%, respectively, while the combined exposure did not affect the grain dry mass.

In agreement with our results, other studies have shown that the application of ZnO NPs could improve plant growth and biomass. According to Zhang et al. [57], the addition of ZnO NPs in Cd-contaminated paddy soil increased rice biomass by 13% to 43%. Seed priming with ZnO NPs combined with sodium selenite and sodium selenate increased rice seed vigor, the crop growth rate, and productivity [58]. 

Similar results were observed for lettuce plants exposed to GSNO combined with CuO NPs. This treatment promoted a threefold increase in the dry weight of lettuce [32]. This indicated that the association of a nitric oxide donor and metallic nanoparticles had a positive effect on plant growth.

### 3.3. Ionomic Profile in Rice Husks and Grains

The effects of the applied treatments on elemental distribution were evaluated by determining their concentration on husks and rice grains (Table S1). Table 2 illustrates the significant changes in the elemental concentration of grains and husks, compared with the corresponding controls. It is important to emphasize that Zn concentrations increased only in the husks of rice plants that were treated with ZnO NPs (+66%) and GSNO–ZnO NPs (+68%). All treated plants showed lower concentrations of K, P, and Cd in husks than in the control samples. Husks from treatments with ZnO NPs and GSNO–ZnO NPs showed lower concentrations of Mg and Fe than those of the control plants. 

GSNO-only treated rice plants showed modifications in the elemental concentration of rice grains. This treatment caused increases in As (+44%) and Fe (+25%) concentrations, compared to the control. Unfortunately, none of the applied treatments caused an increase in Zn in rice grains; rather, in this study’s conditions, this element was not able to accumulate in the grains. However, Zn-enriched rice husks could be applied on soil, during rice cultivation, as a fertilizer.

Bala et al. [59] evaluated the efficacy of the foliar application of ZnO NPs for rice biofortification by applying concentrations (500, 1000 and 5000 µg mL^−1^) higher than ours (100 µg mL^−1^). They reported a slight increase in Zn concentration in the grains 20,280 µg kg^−1^ (+61%) at the highest ZnO NPs treatment. However, they did not clarify whether they had evaluated the grains separated from their respective husks, which may influence a comparison of their reported values to our findings. Moreover, Bala et al. observed that ZnO NPs application caused a decrease in P and Cu and an increase in Mn and Fe concentration in rice grains.

### 3.4. Transport Factor of Minerals from Roots to Shoots

The internal concentrations of metals found in a plant are controlled by balance and by mechanisms that transport metal cations of broad substrate and specificity. In this way, the amount of internal Zn present in the cultivar can affect the concentration of other macro- and microelements that exist there [60]. To evaluate the transport capacity of minerals from roots to shoots, transport factor (TF) was calculated (Table 3). The mean concentrations of elements in root and shoot samples are presented in the Supplementary Materials (Table S2). We found that only macroelement concentrations were disturbed by the foliar application of the treatments described in this study. Sodium, Mg, and K translocation decreased in all groups, compared to the control samples. A decrease in P translocation was observed in samples of the GSNO–ZnO NPs group, and an increase in Ca translocation was observed in samples of the ZnO NPs group. 

In a study of wheat and rice plants, Mazhar et al. [61] reported that the application of ZnO NPs resulted in a salinity balance that induced the translocation of Na or K to the detriment of Zn, promoting an increase or regulation in the elements that were in excess or shortage [61]. Unlike the study of Mazhar et al. [61], this study was performed without any type of stress, which may explain the reason that application of ZnO NPs decreased and regulated the concentration of Na, Mg, P, and K. The results of the present study indicated that the significant decrease in the translocation of Na, Mg, P, and K may be a homeostatic regulation of the rice plant, avoiding further oxidative stress [57,61]. The observed decrease in Na may represent a positive result, since high concentrations of this element can result in homeostatic imbalances and oxidative stress, leading to nutrient deficiency, reduced plant growth, and death [62,63].

### 3.5. Biochemical Parameters 

#### 3.5.1. Enzymatic Activity

**Figure 3:**
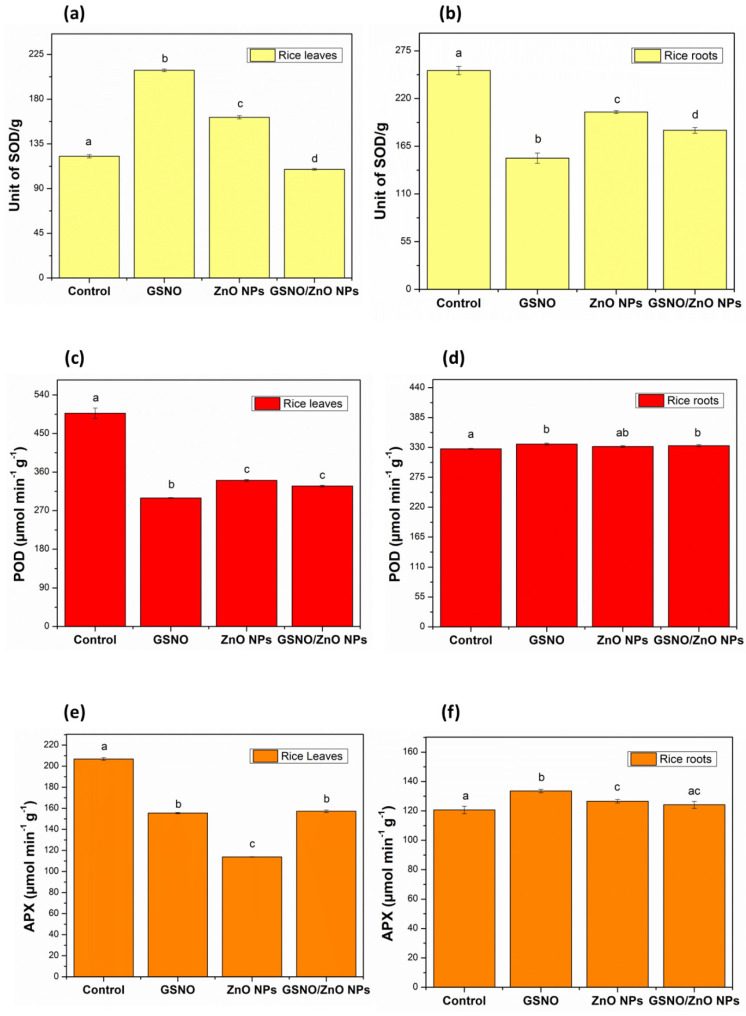
Enzymatic antioxidant activity in rice leaves and roots (pot experiments): (**a**) superoxide dismutase (SOD) in rice leaves; (**b**) superoxide dismutase (SOD) in rice roots; (**c**) peroxidase (POD) in rice leaves; (**d**) peroxidase (POD) in rice roots; (**e**) ascorbate peroxidase (APX) in rice leaves; (**f**) ascorbate peroxidase (APX) in rice roots. Note: different letters indicate statistical differences by Tukey’s test (*p* < 0.05).

Figure 3 illustrates the enzymatic activity of SOD (yellow), POD (red), and APX (orange) in the leaves (Figure 3a,c,e, respectively) and roots (Figure 3b,d,f, respectively) for all treatments, compared to their respective control groups.

These enzymes are crucial for the plant’s defense against abiotic stress, and other metabolites such as glutathione, ascorbic acid, and phenolic compounds could also participate in eliminating ROS [64,65]. 

Regarding the analysis of the leaves, it can be observed that the activity of APX and POD enzymes decreased with the GSNO, ZnO NPs, and GSNO–ZnO NPs treatments, compared to the control group (Figure 3c,d). The low activity of APX and POD in leaves could possibly indicate that the plant did not require high levels of ascorbate peroxidase and peroxidase enzymes to eliminate ROS such as oxygen peroxide. However, SOD activity increased with the GSNO and ZnO NPs treatments, compared to their respective control groups (Figure 3a). This increase in SOD activity could be due to the low elimination rate of ROS by APX and POD enzymes [65,66]. Additionally, the low production of ROS in rice leaves that received the ZnO NPs and GSNO treatments and their combination could justify the low activity of APX and POD as an alternative route for removing oxygen peroxide. In general, the rice leaves exposed to ZnO NPs, GSNO, and GSNO–ZnO NPs possibly produced greater amounts of NO, increasing SOD activity mainly when GSNO–ZnO NPs were applied. With an increase in SOD and a decrease in APX and POD activity, the roots were also affected, in line with data reported by Rolly et al. [67].

Comparing the roots data, as shown in Figure 3f, the activity of the APX enzyme showed a slight increase when the rice plants were treated with ZnO NPs and GSNO separately, compared to the control group, indicating that these treatments did not induce an increase in H_2_O_2_. Corroborating this hypothesis, the POD determination also showed a very small significant increase. However, SOD enzyme activity decreased significantly when the plants were treated with GSNO, ZnO NPs, and GSNO–ZnO NPs, compared to the control group (Figure 3b). This reduction in SOD activity could be related to the moderate need for this enzyme to balance O_2_^•−^ in the roots, which maintain the concentration of H_2_O_2_ near to the control level in plants [67,68]. SODs control the levels of these other species by regulating the concentration of O_2_^•−^. The dismutase activity of SODs speeds up the reaction of superoxide anions (O_2_^•−^) with each other, resulting in the chemical formation of oxygen and hydrogen peroxide (2O_2_^•−^ + 2H^+^ → H_2_O_2_ + O_2_) [69]. As expected for a foliar application, the treatments did not produce a representative change in the enzymatic activity in roots, while in leaves the treatments’ effects were evident.

#### 3.5.2. S-Nitrosothiols and NO_2_^−^ Determinations

**Figure 4:**
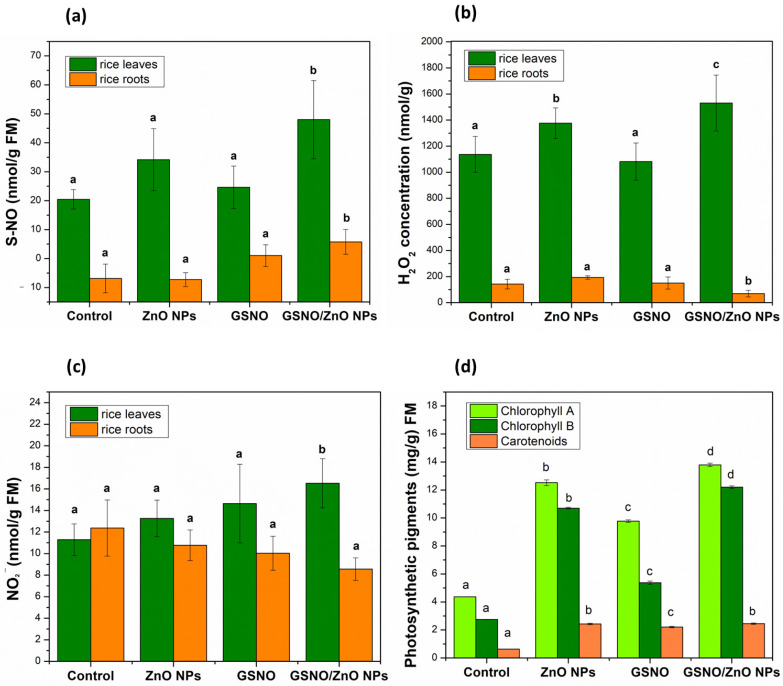
Concentrations (nM mg^−1^ FM) of (**a**) S-nitrosothiols (SNOs), (**b**) hydrogen peroxide H_2_O_2_, and (**c**) nitrite (NO_2_) in rice leaves and roots., (**d**) Concentration of photosynthetic pigments (mg g^−1^ FM) in rice leaves in pot experiments. Note: different letters indicate statistical difference by Tukey’s test (*p* < 0.05). FM: fresh mass.

Figure 4a illustrates the concentration of S-NO (nmol/g FM); Figure 4b and Figure 4c demonstrate, respectively, the concentration of H_2_O_2_ (nmol/g FM) and NO_2_^−^ (nmol/g FM) in the leaves and roots of rice. 

Significant changes were observed in the concentrations of S-NO, H_2_O_2_, and NO_2_^−^ after foliar application of all treatments (Figure 4a–c). The concentration of S-NO showed a significant increase of 135% in the leaves of plants exposed to GSNO–ZnO NPs, compared to the control, and in the other treatments there was no significant increase in S-nitrosothiols (Figure 4a). In the roots, the same pattern was observed, which meant that the concentrations of S-NO showed a significant increase after the foliar application of the GSNO–ZnO NPs treatment.

It was possible to observe that H_2_O_2_ in leaves behaved as S-NO, which meant that the groups containing ZnO NPs alone or combined with GSNO showed the highest levels of this ROS. This was probably a response to the metal stress induced by zinc. This effect may be dose-dependent, as other authors have mentioned that the use of 25 mg L^−1^ of ZnO NPs in mustard plants decreased H_2_O_2_ levels, while the 100 mg L^−1^ concentration (same dose of our study) significantly increased the H_2_O_2_ concentration [70,71]. In roots, the level of H_2_O_2_ showed only a slight decrease after the exposition to GNSO–ZnO NPs, showing that the roots were less affected. 

In the leaves, NO_2_ concentration significantly increased to 17 ± 2.28 nmol g^−1^ FM after the application of GSNO–ZnO NPs. In roots, the concentration of NO_2_^−^ did not change for any treatment applied in relation to the control, similar to what was observed for H_2_O_2_. 

In plants, S-NO is a metabolite related to NO, indicating its relationship with oxidative stress. The increase in S-NO, H_2_O_2_, and NO_2_^−^ concentrations in plants may indicate some toxicity [59]. Molnár et al. [71] reported that the toxicity of ZnO NPs was dependent on the type of plant and material concentration. Kohatsu et al. [70] applied different concentrations of CuO NPs to lettuce seeds and observed an increase in S-NO and NO_2_^−^ levels, dependent on the concentration of NPs. On the other hand, Pelegrino et al. [32] observed a decrease in S-NO concentration in leaves treated with GSNO and CuO NPs and a significant increase in nitrite in lettuce leaves when treated with GSNO. In a trend that was opposite to our results, these authors showed that the application of CuO NPs + GSNO decreased the levels of S-NO in the leaves but increased the levels of S-NO in the roots [32].

#### 3.5.3. Pigment Contents

Photosynthesis is an important indicator of adaptability in plants that are under abiotic and biotic stress—that is, plants under stress would significantly decrease in chlorophyll content in the presence of any toxic material. However, the results shown in Figure 4d illustrate the opposite. After the application of ZnO NPs, the chlorophyll content increased, respectively, by 187% for chlorophyll A and by 290% for chlorophyll B. When GSNO was applied, the chlorophyll A content increased by 124% and the chlorophyll B content increased by 95%. Finally, the application of the two combined treatments (GSNO–ZnO NPs) significantly increased the chlorophyll A content by 216% and the chlorophyll B content by 345%. 

Studies reported that ZnO NPs, when properly applied, can be efficient fertilizers to increase the Zn content in plants, reduce the effect of ROS, enhance the growth of seedlings, and alleviate toxic effects generated by metals such as Cd and As [72,73]. For example, Mazhar et al. [61]. conducted pot experiments with the application of different sources of Zn (ZnSO_4_.7H_2_O, ZnO, and ZnO NPs) in soil for rice and wheat cultivation. They showed that all of the used Zn sources effectively alleviated the negative effects of salt stress on plant growth, yield, and Zn concentration, but the optimal improvements were observed when ZnO NPs were applied. Ahmad et al. [65] showed that the application of ZnO NPs to As-stressed soybean plants resulted in a considerable increase in the lengths of shoots and roots, a net photosynthetic rate, transpiration, stomatal conductance, and photochemical yield. Zhang et al. [57]. reported that ZnO NPs could improve plant growth by soil application, especially in the early-growth stage, and alleviate the toxic effects of Cd in rice plants. However, other studies reported negative effects, such as an excess production of ROS and an accumulation or excess of chlorophyll content [72,73,74]. In contrast to our results, Chen et al. [72] demonstrated that the use of 100 mg L^−1^ of ZnO NPs reduced the chlorophyll A content and the chlorophyll B content by 76.7% and 75.7%, respectively. 

A possible explanation for the reduction in chlorophyll content can be the accumulation of Zn in rice leaves and competition with the element Mg, which is significantly involved in the catalysis and insertion of Mg-chelatase into protoporphyrin in chlorophyll synthesis [72,75]. However, our data showed no accumulation of Zn in leaves, which may indicate a positive influence on pigment production. 

Other authors mentioned the positive effects of the use of ZnO NPs on photosynthetic pigments. Song et al. [76] reported increased contents of chlorophyll A and chlorophyll B after the foliar application of ZnO NPs in rice plants, indicating decreased chlorosis and stress under cold temperatures. Another study demonstrated the increased content of chlorophyll A, chlorophyll B, and carotenoids in *Leucaena Leucocephala* plants treated with ZnO NPs [77]. Yan et al. [78] demonstrated that the use of 1–100 mg L^−1^ of ZnO NPs was able to increase, gradually, the content of chlorophyll A and chlorophyll B in rice sprouts. 

The exogenous use of GSNO as a NO donor contributed positively to the increase in the content of photosynthetic pigments in rice. Other authors applied GSNO to heat-stressed rice plants and obtained a significant increase in the content of chlorophyll A, chlorophyll B, and carotenoids. The increase in photosynthetic activity with the application of exogenous GSNO contributed to the protection of proteins found in the chloroplast, leading to better performance of enzymes, such as glutamine synthetase and NADPH-dependent glyceraldehyde-3-phosphate dehydrogenase, as well as improvement on energy transducer system in thylakoids present in chloroplasts [79].

## 4. Conclusions

The microelement zinc is essential for the growth and development of plants, as well as for cellular processes, the production of leaves and grains, and the process of photosynthesis. The foliar application of ZnO NPs and GSNO–ZnO NPs seems to have helped to increase the concentration of zinc only in the husk of rice grains. However, the foliar application of free GSNO led to increases in As and Fe in the grains of the cultivars. Although the Zn concentration did not increase in the grains, there was still the possibility of using rice husks as a possible fertilizer. Rice husks can be converted into ashes, biochar, or vermicompost that can be reapplied in crop fields, as soil amendment can be a sustainable approach in rice production [80].

The enzymatic activity tests indicated that, compared with the control group, the GSNO, ZnO NPs, and GSNO–ZnO NPs treatments decreased the activity of the POD and APX enzymes in the leaves, possibly because they do not require a great elimination of ROS such as H_2_O_2_. The increased activity of enzymes such as SOD in the presence of GSNO and ZnO NPs applications may suggest an attempt to balance the low elimination of ROS by POD and APX. 

All applied materials were able to significantly increase the rate of photosynthetic pigments (chlorophyll A, chlorophyll B, and carotenoids) in cultivars, indicating that the application of 100 µg mL^−1^ of ZnO NPs and 100 mM of GSNO enabled a more than 100% increase in all photosynthetic pigments, compared with their control groups, in the absence of biotic or abiotic stresses. Together, the data obtained in this study indicate that the reduction in macroelements observed by the translocation factor (TF) and the decrease in enzymes such as APX were not enough to unbalance and interfere with the vital processes of rice plants, such as photosynthesis, leaf growth, grain yield, and roots growth. 

In the future, it will be crucial to encourage further exploration into the influence of particle size, concentration, and application routes of ZnO NPs associated with GSNO in rice. Further studies are required to evaluate the impact of these materials under field conditions and their fate in the environment. The molecular and genetic impacts of these materials on rice plants should be assessed.

## Figures and Tables

**Table 1:** Effect of foliar application of ZnO NPs, GSNO, and GSNO–ZnO NPs on the shoot, root, and grain parameters of rice.

Parameter	Control	ZnO NPs	GSNO	GSNO–ZnO NPs
Panicles (n°)	6 ± 0.4 ^a^	6 ± 0.4 ^a^	15.5 ± 2.1 ^b^	11 ± 1 ^c^
Root dry mass (g)	30 ± 2.7 ^a^	30 ± 2 ^a^	25 ± 2.5 ^a^	27 ± 0.6 ^a^
Shoot dry mass (g)	24 ± 1.3 ^a^	24 ± 0.7 ^a^	19 ± 1.8 ^b^	21 ± 1.1 ^b^
Grain dry mass (g)	20 ± 3.1 ^a^	29 ± 1.4 ^b^	27 ± 1.8 ^b^	21 ± 2.3 ^a^

Means within the sub-factor followed by the same letter in a line are not significantly different at *p* ≤ 0.05, according to pair-wise comparison of least square means. Average ± standard error from quadruplicate sample.

**Table 2:** Ionomic profile in rice husks and grains treated with ZnO NPs, GSNO, and GSNO–ZnO NPs. Mean significant increments compared to the control signalized by (+) and reductions (−), and unaffected (equal symbol) (n = 4).

Elements		Grains			Husks		
		ZnO NPs	GSNO	GSNO- ZnO NPs	ZnO NPs	GSNO	GSNO- ZnO NPs
Macroelements	Ca	=	=	=	=	=	=
	K	=	=	=	−27%	−22.8%	−28.5%
	Mg	=	=	=	−41.9%	=	−37.1%
	Na	=	=	=	=	=	=
	P	=	=	=	−47%	−44.9	−63%
Trace elements	As	=	+44%	=	=	=	=
	Cd	=	=	=	−55%	−72%	−52%
	Cr	=	=	=	=	=	=
	Cu	=	=	=	=	=	=
	Fe	=	+25%	=	−24%	=	−26%
	Ni	=	=	=	=	=	=
	Mn	=	=	=	=	=	=
	Pb	=	=	=	=	=	=
	Co	=	=	=	=	=	=
	Zn	=	=	=	+66%	=	+68%

**Table 3:** Translocation factor of rice exposed to different treatments (mean ± SE, n = 4).

Element	Control	ZnO NPs	GSNO	GSNO + ZnO NPs
	Translocation Factor (TF)			
Na	7.7 ± 3.2 ^a^	0.92 ± 0.38 ^b^	1.3 ± 0.7 ^b^	1.7 ± 0.7 ^b^
Mg	5.3 ± 1.5 ^a^	3.6 ± 0.9 ^b^	2.1 ± 0.5 ^b^	1.8 ± 0.4 ^b^
P	2.7 ± 0.5 ^a^	3 ± 1.5 ^a^	1.5 ± 0.7 ^ab^	0.74 ± 0.16 ^b^
K	82 ± 17 ^a^	22 ± 14 ^b^	20 ± 12 ^b^	36 ± 10 ^b^
Ca	0.39 ± 0.11 ^a^	0.66 ± 0.38 ^b^	0.25 ± 0.08 ^a^	0.23 ± 0.12 ^a^
Cr	0.032 ± 0.020 ^a^	0.013 ± 0.005 ^a^	0.023 ± 0.013 ^a^	0.015 ± 0.006 ^a^
Mn	1.2 ± 0.7 ^a^	1 ± 0.5 ^a^	0.94 ± 0.21 ^a^	0.60 ± 0.27 ^a^
Fe	0.004 ± 0.001 ^a^	0.003 ± 0.001 ^a^	0.002 ± 0.002 ^a^	0.002 ± 0.001 ^a^
Co	0.051 ± 0.024 ^a^	0.039 ± 0.017 ^a^	0.052 ± 0.033 ^a^	0.064 ± 0.008 ^a^
Ni	0.082 ± 0.087 ^a^	0.024 ± 0.009 ^a^	0.014 ± 0.002 ^a^	0.017 ± 0.006 ^a^
Cu	0.195 ± 0.027 ^a^	0.16 ± 0.04 ^a^	0.15 ± 0.06 ^a^	0.13 ± 0.05 ^a^
Zn	1.2 ± 0.5 ^a^	1.5 ± 0.8 ^a^	1.1 ± 0.1 ^a^	1.1 ± 0.4 ^a^
As	0.021 ± 0.005 ^a^	0.027 ± 0.007 ^a^	0.012 ± 0.007 ^a^	0.014 ± 0.015 ^a^
Cd	0.462 ± 0.470 ^a^	0.047 ± 0.048 ^a^	0.15 ± 0.09 ^a^	0.16 ± 0.13 ^a^
Pb	0.005 ± 0.004 ^a^	0.0025 ± 0.0003 ^a^	0.002 ± 0.001 ^a^	0.0017 ± 0.0005 ^a^

Means within the sub-factor followed by the same letter in a column are not significantly different at *p* ≤ 0.05, according to pair-wise comparison of least square means.

## Data Availability

Data is contained within the article.

## Supplementary Materials

The following supporting information can be downloaded at: https://www.mdpi.com/article/10.3390/antiox12101871/s1, Table S1: Elemental concentration of ZnO NPs, GSNO and ZnO NPs + GSNO in rice husks and grains (µg kg^−1^), n = 4. Table S2: Elemental concentration of ZnO NPs, GSNO and ZnO NPs + GSNO in rice roots and shoots (µg kg^−1^), n = 4.

## References

[1] Trijatmiko K.R., Dueñas C., Tsakirpaloglou N., Torrizo L., Arines F.M., Adeva C., Balingong J., Oliva N., Sapasap M.V., Borrero J. (2016). Biofortified indica rice attains iron and zinc nutrition dietary targets in the field. Sci. Rep..

[2] Bakhat H.F., Zia Z., Fahad S., Abbas S., Hammad H.M., Shahzad A.N., Abbas F., Alharby H., Shahid M. (2017). Arsenic uptake, accumulation and toxicity in rice plants: Possible remedies for its detoxification: A review. Environ. Sci. Pollut. Res..

[3] Alloway B.J. (2009). Soil factors associated with zinc deficiency in crops and humans. Environ. Geochem. Health.

[4] Hotz C., Brown K.H. (2004). Assessment of the risk of zinc deficiency in populations and options for its control. Food Nutr. Bull..

[5] Younas N., Fatima I., Ahmad I.A., Ayyaz M.K. (2023). Alleviation of zinc deficiency in plants and humans through an effective technique; biofortification: A detailed review. Acta Ecol. Sin..

[6] Shraim A.M. (2017). Rice is a potential dietary source of not only arsenic but also other toxic elements like lead and chromium. Arab. J. Chem..

[7] Bandyopadhyay B., Ray P., Padua S., Ramachandran S., Jena R.K., Deb Roy P., Dutta D.P., Ray S.K. (2018). Priority Zoning of Available Micronutrients in the Soils of Agroecological Sub-regions of North-East India Using Geo-spatial Techniques. Agric. Res..

[8] Phattarakul N., Rerkasem B., Li L.J., Wu L.H., Zou C.Q., Ram H., Sohu V.S., Kang B.S., Surek H., Kalayci M. (2012). Biofortification of rice grain with zinc through zinc fertilization in different countries. Plant Soil.

[9] Dapkekar A., Deshpande P., Oak M.D., Paknikar K.M., Rajwade J.M. (2018). Zinc use efficiency is enhanced in wheat through nanofertilization. Sci. Rep..

[10] Mejia L.A., Dary O., Boukerdenna H. (2017). Global regulatory framework for production and marketing of crops biofortified with vitamins and minerals. Ann. N. Y. Acad. Sci..

[11] Wang Q., Liu S., Wang H., Yang Y. (2016). In situ pore-forming alginate hydrogel beads loaded with in situ formed nano-silver and their catalytic activity. Phys. Chem..

[12] Yang C.C., Mai Y.W. (2014). Thermodynamics at the nanoscale: A new approach to the investigation of unique physicochemical properties of nanomaterials. Mater. Sci. Eng. R Rep..

[13] Prasad R., Bhattacharyya A., Nguyen Q.D. (2017). Nanotechnology in sustainable agriculture: Recent developments, challenges, and perspectives. Front. Microbiol..

[14] Taheri M., Hania A.Q., Alimohammad A.Q., Yoosefi M. (2015). The Effects of Zinc-Oxide Nanoparticles on Growth Parameters of Corn (SC704). STEM Fellowsh. J..

[15] Abdallah Y., Liu M., Ogunyemi S.O., Ahmed T., Fouad H., Abdelazez A., Yan C., Yang Y., Chen J., Li B. (2020). Bioinspired Green Synthesis of Chitosan and Zinc Oxide Nanoparticles with Strong Antibacterial Activity against Rice Pathogen *Xanthomonas oryzae* pv. oryzae. Molecules.

[16] Thounaojam T.C., Meetei T.T., Devi Y.B., Panda S.K., Upadhyaya H. (2021). Zinc oxide nanoparticles (ZnO-NPs): A promising nanoparticle in renovating plant science. Acta Physiol. Plant..

[17] Khot L.R., Sankaran S., Maja J.M., Ehsani R., Schuster E.W. (2012). Applications of nanomaterials in agricultural production and crop protection: A review. Crop Prot..

[18] Capaldi Arruda S.C., Diniz Silva A.L., Moretto Galazzi R., Antunes Azevedo R., Zezzi Arruda M.A. (2015). Nanoparticles applied to plant science: A review. Talanta.

[19] Farnese F.S., Menezes-Silva P.E., Gusman G.S., Oliveira J.A. (2016). When bad guys become good ones: The key role of reactive oxygen species and nitric oxide in the plant responses to abiotic stress. Front. Plant Sci..

[20] Santisree P., Bhatnagar-Mathur P., Sharma K.K. (2015). NO to drought-multifunctional role of nitric oxide in plant drought: Do we have all the answers?. Plant Sci..

[21] Seabra A.B., Pasquôto T., Ferrarini A.C.F., Santos M.C., Haddad P.S., de Lima R. (2014). Preparation, characterization, cytotoxicity, and genotoxicity evaluations of thiolatedand s-nitrosated superparamagnetic iron oxide nanoparticles: Implications for cancer treatment. Chem. Res. Toxicol..

[22] Corpas F.J., Barroso J.B. (2015). Nitric oxide from a “green” perspective. Nitric Oxide.

[23] Seabra A.B., Rai M., Durán N. (2014). Nano carriers for nitric oxide delivery and its potential applications in plant physiological process: A mini review. J. Plant Biochem. Biotechnol..

[24] Simontacchi M., Galatro A., Ramos-Artuso F., Santa-María G.E. (2015). Plant Survival in a Changing Environment: The Role of Nitric Oxide in Plant Responses to Abiotic Stress. Front. Plant Sci..

[25] Filho P.C., Serra O.A. (2015). Liquid phase synthesis methodologies for the obtainment of rare earth-based inorganic nanomaterials. Química Nova.

[26] Madhumitha G., Elango G., Roopan S.M. (2016). Biotechnological aspects of ZnO nanoparticles: Overview on synthesis and its applications. Appl. Microbiol. Biotechnol..

[27] Yoko A., Gimyeong S., Tomai T., Adschiri T. (2020). Synthesis of Nanoparticles Using Supercritical Water: Process Design, Surface Control, and Nanohybrid Materials. KONA Powder Part. J..

[28] Riman R. (2002). Cristallisation hydrothermale de ceramiques. Ann. De Chim. Sci. Des Matériaux.

[29] Basnet P., Chatterjee S. (2020). Structure-directing property and growth mechanism induced by capping agents in nanostructured ZnO during hydrothermal synthesis—A systematic review. Nano-Struct. Nano-Objects.

[30] Mayedwa N., Mongwaketsi N., Khamlich S., Kaviyarasu K., Matinise N., Maaza M. (2018). Green synthesis of zin tin oxide (ZnSnO_3_) nanoparticles using *Aspalathus Linearis* natural extracts: Structural, morphological, optical and electrochemistry study. Appl. Surf. Sci..

[31] Marinho J.Z., Romeiro F.C., Lemos S.C.S., Motta F.V., Riccardi C.S., Li M.S., Longo E., Lima R.C. (2012). Urea-Based Synthesis of Zinc Oxide Nanostructures at Low Temperature. J. Nanomater..

[32] Pelegrino M.T., Pieretti J.C., Lange C.N., Kohatsu M.Y., Freire B.M., Batista B.L., Seabra A.B. (2021). Foliar spray application of CuO nanoparticles (NPs) and S-nitrosoglutathione enhances productivity, physiological and biochemical parameters of lettuce plants. J. Chem Technol. Biotechnol..

[33] Pelegrino M.T., Kohatsu M.Y., Seabra A.B., Monteiro L.R., Gomes D.G., Oliveira H.C., Rolim W.R., de Jesus T.A., Batista B.L., Lange C.N. (2020). Effects of copper oxide nanoparticles on growth of lettuce (*Lactuca sativa* L.) seedlings and possible implications of nitric oxide in their antioxidative defense. Environ. Monit. Assess..

[34] Shams M., Ekinci M., Turan M., Dursun A., Kul R., Yildirim E. (2019). Growth, nutrient uptake and enzyme activity response of lettuce (*Lactuca sativa* L.) to excess copper. J. Environ. Sustain..

[35] Liu S., Dong Y., Xu L., Kong J. (2014). Effects of foliar applications of nitric oxide and salicylic acid on salt-induced changes in photosynthesis and antioxidative metabolism of cotton seedlings. Plant Growth Regul..

[36] Anderson M.D., Prasad T.K., Stewart C.R. (1995). Changes in isozyme profiles of catalase, peroxidase, and glutathione reductase during acclimation to chilling in mesocotyls of maize seedlings. Plant Physiol..

[37] Oliveira H.C., Gomes B.C., Pelegrino M.T., Seabra A.B. (2016). Nitric oxide releasing chitosan nanoparticles alleviate the effects of salt stress in maize plants. Nitric Oxide.

[38] Lichtenthaler H.K., Wellburn A.R. (1983). Determinations of total carotenoids and chlorophylls a and b of leaf extracts in different solvents. Biochem. Soc. Trans..

[39] Paniz F., Pedron T., Moreira B.F., Torres D.P., da Silva F.F., Lemos B.B. (2018). Effective procedures for the determination of As, Cd, Cu, Fe, Hg, Mg, Mn, Ni, Pb, Se, Th, Zn, U and rare earth elements in plant and foodstuffs. Anal. Methods.

[40] Aneesh P., Vanaja K., Jayaraj M. Synthesis of ZnO Nanoparticles by Hydrothermal Method. Proceedings of the SPIE, Nanophotonic Materials IV.

[41] Bharti D.B., Bharati A.V. (2016). Synthesis of ZnO nanoparticles using a hydrothermal method and a study its optical activity. Luminescence.

[42] Ismail A.A., El-Midany T.A., Abdel-Aal E.A., El-Shall H. (2005). Application of statistical design to optimize the preparation of ZnO nanoparticles via hydrothermal technique. Mater. Lett..

[43] Søndergaard T., Bozhevolnyi S., Beermann J., Novikov S., Devaux E., Ebbesen T. (2011). Extraordinary optical transmission with tapered slits: Effect of higher diffraction and slit resonance orders. J. Opt. Soc. Am..

[44] Rai P., Yu Y.T. (2012). Citrate-assisted hydrothermal synthesis of single crystalline ZnO nanoparticles for gas sensor application. Sens. Actuators B.

[45] Vlazan P., Ursu D.H., Irina-Moisescu C., Miron I., Sfirloaga P., Rusu E. (2015). Structural and electrical properties of TiO_2_/ZnO core–shell nanoparticles synthesized by hydrothermal method. Mater. Charact..

[46] Droepenu E.K., Wee B.S., Chin S.F., Kok K.Y., Maligan M.F. (2022). Zinc Oxide Nanoparticles Synthesis Methods and its Effect on Morphology: A Review. Biointerface Res. Appl. Chem..

[47] Alver U., Tanriverdi A., Akgul O. (2016). Hydrothermal preparation of ZnO electrodes synthesized from different precursors for electrochemical supercapacitors. Synth. Met..

[48] Wojnarowicz J., Chudoba T., Lojkowski W. (2020). A Review of Microwave Synthesis of Zinc Oxide Nanomaterials: Reactants, Process Parameters and Morphologies. Nanomaterials.

[49] Perillo P.M., Atia M.N., Rodríguez D.F. (2018). Studies on the growth control of ZnO nanostructures synthesized by the chemical method. Rev. Matéria.

[50] El-Ghazzawy E.H., Zakaly H.M., Alrowaily A.W., Saafan S.A., Ene A., Abo-aita N.M., Atlam A.S. (2023). Delving into the properties of nanostructured Mg ferrite and PEG composites: A comparative study on structure, electrical conductivity, and dielectric relaxation. Heliyon.

[51] Freire B.M., Paniz F.P., Lange C.N., Pedron T., da Silva J.T., Sanchez F.S., Parfitt J.M.B., Batista B.L. (2023). Effect of water management on human exposure to inorganic arsenic and other trace elements through rice consumption. J. Food Compos. Anal..

[52] Bao Y., Pan C., Liu W., Li Y., Ma C., Xing B. (2019). Iron plaque reduces cerium uptake and translocation in rice seedlings (*Oryza sativa* L.) exposed to CeO_2_ nanoparticles with different sizes. Sci. Total Environ..

[53] Zhou Y., Liu X., Yang X., Du Laing G., Yang Y., Tack F.M., Bundschuh J. (2023). Effects of platinum nanoparticles on rice seedlings (*Oryza sativa* L.): Size-dependent accumulation, transformation, and ionomic influence. Environ. Sci. Technol..

[54] Aqeel U., Aftab T., Khan M.M.A., Naeem M., Khan M.N. (2022). A comprehensive review of impacts of diverse nanoparticles on growth, development and physiological adjustments in plants under changing environment. Chemosphere.

[55] Hong J., Wang C., Wagner D.C., Gardea-Torresdey J.L., He F., Rico C.M. (2021). Foliar application of nanoparticles: Mechanisms of absorption, transfer, and multiple impacts. Environ. Sci. Nano.

[56] Wang Y., Deng C., Rawat S., Cota-Ruiz K., Medina-Velo I., Gardea-Torresdey J.L. (2021). Evaluation of the effects of nanomaterials on rice (*Oryza sativa* L.) responses: Underlining the benefits of nanotechnology for agricultural applications. ACS Agric. Sci..

[57] Zhang W., Long J., Li J., Zhang M., Xiao G., Ye X., Zeng H. (2019). Impact of ZnO nanoparticles on Cd toxicity and bioaccumulation in rice (*Oryza sativa* L.). Environ. Sci. Pollut. Res..

[58] Adhikary S., Biswas B., Chakraborty D., Timsina J., Pal S., Chandra Tarafdar J., Roy S. (2022). Seed priming with selenium and zinc nanoparticles modifies germination, growth, and yield of direct-seeded rice (*Oryza sativa* L.). Sci. Rep..

[59] Bala R., Kalia A., Dhaliwal S.S. (2019). Evaluation of Efficacy of ZnO Nanoparticles as Remedial Zinc Nanofertilizer for Rice. J. Soil Sci. Plant Nutr..

[60] Nakandalage N., Nicolas M., Norton R.M., Hirotsu N., Milham P.J., Seneweera S. (2016). Improving Rice Zinc Biofortification Success Rates Through Genetic and Crop Management Approaches in a Changing Environment. Front. Plant Sci..

[61] Mazhar Z., Akhtar J., Alhodaib A., Naz T., Zafar M.I., Iqbal M.M., Fatima H., Naz I. (2022). Efficacy of ZnO nanoparticles in Zn fortification and partitioning of wheat and rice grains under salt stress. Sci. Rep..

[62] Gupta R.K., Singh R.R., Tanji K.K. (1990). Phosphorus release in sodium ion dominated soils. Soil Sci. Soc. Am. J..

[63] Yousfi S., Wissal M., Mahmoudi H., Abdelly C., Gharsalli M. (2007). Effect of salt on physiological responses of barley to iron deficiency. Plant Physiol. Biochem..

[64] Abdel Latef A.A.H., Alhmad M.F.A., Abdelfattah K.E. (2017). The Possible Roles of Priming with ZnO Nanoparticles in Mitigation of Salinity Stress in Lupine (*Lupinus termis*) Plants. J. Plant Growth Regul..

[65] Ahmad P., Alyemeni M.N., Al-Huqail A.A., Alqahtani M.A., Wijaya L., Ashraf M., Kaya C., Bajguz A. (2020). Zinc Oxide Nanoparticles Application Alleviates Arsenic (As) Toxicity in Soybean Plants by Restricting the Uptake of as and Modulating Key Biochemical Attributes, Antioxidant Enzymes, Ascorbate-Glutathione Cycle and Glyoxalase System. Plants.

[66] Hajiboland R., Ahmad P. (2014). Reactive oxygen species and photosynthesis. Oxidative Damage to Plants.

[67] Rolly N.K., Lee S.-U., Imran Q.M., Hussain A., Mun B.-G., Kim K.-M., Yun B.-W. (2019). Nitrosative stress-mediated inhibition of OsDHODH1 gene expression suggests roots growth reduction in rice (*Oryza sativa* L.). Biotech.

[68] Upadhyaya H., Roy H., Shome S., Tewari S., Bhattacharya M.K., Panda S.K. (2017). Physiological impact of Zinc nanoparticle on germination of rice (*Oryza sativa* L.) seed. J. Plant Sci. Phytopathol..

[69] Wang Y., Branicky R., Noë A., Hekimi S. (2018). Superoxide dismutases: Dual roles in controlling ROS damage and regulating ROS signaling. J. Cell Biol..

[70] Kohatsu M.Y. (2021). Efeitos de Nanopartículas Biogênicas de Óxido de Cobre em Bioensaios in vitro e in vivo com Alface (*Lactuca sativa* L.). Master’s Thesis.

[71] Molnár A., Ronavari A., Belteky P., Szollosia R., Valyon E., Olaha D., Razga Z., Ordog A., Konya Z., Kolbert Z. (2020). ZnO nanoparticles induce cell wall remodeling and modify ROS/RNS signalling in roots of Brassica seedlings. Ecotoxicol. Environ. Saf..

[72] Chen J., Dou R., Yang Z., You T., Gao X., Wang L. (2018). Phytotoxicity and bioaccumulation of zinc oxide nanoparticles in rice (*Oryza sativa* L.). Plant Physiol. Biochem..

[73] Pan L., Zhao L., Jiang W., Wang M., Chen X., Shen X., Yin C., Mao Z. (2022). Effect of Zinc Oxide Nanoparticles on the Growth of Malus hupehensis Rehd. Seedl. Front. Environ. Sci..

[74] Lin Y.-P., Wu M.-C., Charng Y.Y. (2016). Identification of a Chlorophyll Dephytylase Involved in Chlorophyll Turnover in Arabidopsis. Plant Cell.

[75] Luo M., Weinstein J.D., Walker C.J. (1999). Magnesium chelatase subunit D from pea: Characterization of the cDNA, heterologous expression of an enzymatically active protein and immunoassay of the native protein. Plant Mol. Biol..

[76] Song Y., Jiang M., Zhang H., Li R. (2021). Zinc Oxide Nanoparticles Alleviate Chilling Stress in Rice (*Oryza sativa* L.) by Regulating Antioxidative System and Chilling Response Transcription Factors. Molecules.

[77] Venkatachalam P., Jayaraj M., Manikandan R., Geetha N., Rene E.R., Sharma N.C., Sahi S.V. (2017). Zinc oxide nanoparticles (ZnONPs) alleviate heavy metal-induced toxicity in *Leucaena leucocephala* seedlings: A physiochemical analysis. Plant Physiol. Biochem..

[78] Yan S., Wu F., Zhou S., Yang J., Tang X., Ye W. (2021). Zinc oxide nanoparticles alleviate the arsenic toxicity and decrease the accumulation of arsenic in rice (*Oryza sativa* L.). BMC Plant Biol..

[79] Song L., Yue L., Zhao H., Hou M. (2013). Protection effect of nitric oxide on photosynthesis in rice under heat stress. Acta Physiol. Plant.

[80] Karam D.S., Nagabovanalli P., Rajoo K.S., Ishak C.F., Abdu A., Rosli Z., Zulperi D. (2022). An overview on the preparation of rice husk biochar, factors affecting its properties, and its agriculture application. J. Saudi Soc. Agric. Sci..

