# Supplementary material for: Implications of ZnO Nanoparticles and S-Nitrosoglutathione on Nitric Oxide, Reactive Oxidative Species, Photosynthetic Pigments, and Ionomic Profile in Rice
Source: Antioxidants (Basel). 2023 Oct 17;12(10):1871. doi: 10.3390/antiox12101871 (PMC10604056; doi:10.3390/antiox12101871)
Supplement: Supplementary file 1 [file antioxidants-12-01871-s001.zip › antioxidants-2647967-supplementary.pdf]

Supplementary material

Table S1 Elemental concentration of ZnO NPs, GSNO and ZnO NPs + GSNO in rice husks and grains (µg kg^{-1}), n=4.

	Grain		Husk		Grain		Husk	
	Control				GSNO			
	Mean	Error	Mean	Error	Mean	Error	Mean	Error
Na	59501	17365	1641410	350117.8	48950	6059	1020258	178495
Mg	1288939	61628	808558	42141.3	1262285	21704	614826	52212
P	4182826	173251	1183496	171867.3	4185603	69017	651794	82401
K	3073680	144689	8315633	269122.0	3187861	32629	6415822	443682
Ca	96694	7799	565796	43211.5	99935	1413	504356	39808
Cr	38	10.5	680	80.1	23	1.42	596	27.4
Mn	13453	966.9	50180	3743.0	13074	908.79	36882	5890.2
Fe	6562	527.9	15001	1128.6	8213	367.12	12956	360.4
Co	5	0.4	27	1.7	5	0.26	31	1.2
Ni	60	2.7	661	68.2	57	3.31	747	60.9
Cu	2365	190.4	2374	164.1	2160	62.17	2838	283.3
Zn	20184	757.2	21191	392.1	19585	1109.93	23393	1454.0
As	89	8.8	119	4.9	130	6.20	146	7.9
Cd	33	10.7	27	2.2	23	4.59	8	2.7
Pb	152	15.0	28	2.0	92	38.17	41	4.7

	Grain		Husk		Grain		Husk	
	ZnO NPs				ZnO NPs + GSNO			
	Mean	Error	Mean	Error	Mean	Error	Mean	Error
Na	77659	16256	1323540	366868	35116	4754	595354	73151
Mg	1197117	35406	470155	80227	1268028	25285	508405	39432
P	4126376	105973	618284	81963	4176406	97827	435163	29955
K	2990985	52861	6066113	317863	3153602	96778	5948433	341589
Ca	98125	2137	457882	10426	102769	5316	486856	24437
Cr	32	4.7	473	54.0	32	4.24	568	62.7
Mn	14063	1463.0	46623	7571.9	16862	595.43	56047	3704.2
Fe	6818	84.0	11377	681.6	7636	208.92	11106	568.4
Co	4	0.2	23	0.2	6	0.43	27	1.6
Ni	76	4.5	733	87.8	71	5.16	722	32.9
Cu	1773	187.3	2421	196.3	1755	541.94	2187	250.1
Zn	19420	788.1	35272	2191.4	20957	974.31	35779	3675.8
As	107	6.9	128	9.1	110	7.30	106	2.9
Cd	39	6.4	12	1.7	20	3.22	13	4.4
Pb	283	92.5	20	3.8	83	24.28	21	1.4

Table S2 Elemental concentration of ZnO NPs, GSNO and ZnO NPs + GSNO in rice roots and shoots (μg kg^{-1}), n=4.

	Roots		Shoots		Roots		Shoots	
	Control				GSNO			
	Mean	Error	Mean	Error	Mean	Error	Mean	Error
Na	393465	276045	1502384	1431180	1258919	643348	1138695	921456
Mg	684567	221363	3020752	785389	802672	118480	1951225	539578
P	386069	205120	626388	406576	622901	378453	629576	368025
K	393443	142131	22421711	13413304	1104432	572130	22443278	2418901
Ca	3363205	965941	905073	523197	3646350	691843	993521	348187
Cr	6044	4474	106	34	4314	1436	103	52
Mn	67181	40424	44399	28266	89865	22872	71956	28144
Fe	27248834	15006591	63612	51930	32651303	4971352	45895	55243
Co	950	219	47	14	1034	185	42	21
Ni	2035	618	107	89	2093	614	37	14
Cu	3942	973	694	183	3233	959	337	136
Zn	35721	10505	31632	6566	34636	8391	32846	12518
As	17847	3991	341	100	15275	3211	150	88
Cd	1167	445	53	36	848	328	44	32
Pb	108	67	27	24	113	58	15	11
	Roots		Shoots		Roots		Shoots	
	ZnO NPs				ZnO NPs + GSNO			
	Mean	Error	Mean	Error	Mean	Error	Mean	Error
Na	547254	182491	652170	393878	681651	163913	1539220	825257
Mg	586859	153046	2239802	330118	805358	128904	1694505	569892
P	441389	234167	970482	524416	446306	50964	482824	357615
K	813061	467813	17670400	3934239	705563	138798	21871005	7165888
Ca	2563901	1152645	1117149	378817	3397272	779651	897334	348792
Cr	5362	2147	64	9	6093	1652	87	50
Mn	71167	39863	53077	21275	80219	27153	59950	29488
Fe	24142633	5408367	61571	26762	24888407	4616985	53266	12821
Co	1095	463	48	17	964	100	56	11
Ni	1773	734	47	20	2361	498	61	46
Cu	5352	1847	786	498	5118	1643	570	338
Zn	43471	27540	39997	11624	43057	10312	40457	11559
As	9214	3185	315	113	14921	4544	180	91
Cd	1092	339	52	26	1159	206	40	49
Pb	704	883	29	20	199	131	21	14
